# Which patient reported outcome domains are important to the rheumatologists while assessing patients with rheumatoid arthritis?

**DOI:** 10.1186/s41927-019-0087-2

**Published:** 2019-09-05

**Authors:** Aprajita Jagpal, Ronan O’Beirne, Melanie S. Morris, Bernadette Johnson, James Willig, Huifeng Yun, Andrea L. Cherrington, Liana Fraenkel, Jeffrey R. Curtis, Monika M. Safford, Iris Navarro-Millán

**Affiliations:** 1 University of Alabama at Birmingham, Birmingham, AL USA; 2 Yale University, New Haven, CT USA; 3 Division of General Internal Medicine, Weill Cornell Medicine, 420 E 70th St., LH-363, New York, NY 10021 USA; 4 Division of Rheumatology, Hospital for Special Surgery, 420 E 70th St., LH-363, New York, NY 10021 USA

**Keywords:** Rheumatoid arthritis, Shared decision, Physician perspective, Patient reported outcomes

## Abstract

**Background:**

Patient-reported outcomes (PROs) aid in rheumatoid arthritis (RA) management, but it is not well understood which measures would be most relevant to the rheumatologists for making treatment decisions.

**Methods:**

We recruited rheumatologists nationally to participate in moderated structured group teleconference discussions using the nominal group technique. Participants in each group generated lists of the elements from patient’s history and signs that they use to make treatment recommendations for RA. Each participant then selected the three most important elements from the generated list. The results of each group were then combined and summarized.

**Results:**

Twenty-five rheumatologists participated in 4 groups (group size ranged from 4 to 8) and 150 available ranking votes across all groups. The statements generated across the 4 groups were categorized into 13 topics (including symptoms, physical function, comorbidities, social aspects, physical findings, response to treatment, treatment adherence, pain management, side effects, tests, access to care, contraception, and organ involvement), 10 of which received ranking votes. Symptoms received the highest ranking (46% of votes), followed by physical function (16%), and physical findings (13%). Among the unranked topics, social aspects had the highest number of statements (8 statements).

**Conclusion:**

Rheumatologists highly valued patient-reported RA symptoms and physical function to inform their treatment decisions, even above objective data such as physical findings and test results. These results can guide the selection of validated PRO measures to assess these domains to inform the clinical care of patients with rheumatoid arthritis.

**Electronic supplementary material:**

The online version of this article (10.1186/s41927-019-0087-2) contains supplementary material, which is available to authorized users.

## Background

Determining the best therapeutic approach for patients with rheumatoid arthritis (RA) requires disease activity monitoring at each patient visit [1]. Besides physician-assessed disease activity indices, patient-reported outcomes (PROs) should be incorporated to assess a patient’s health [2]. It remains unclear which specific instrument would be helpful for the rheumatologist in making therapeutic recommendations for their patients with RA and in providing holistic care. This is despite having multiple options of PROs to choose from such as the Health Assessment Questionnaire (HAQ), Routine Assessment of Patient Index Data 3 (RAPID3), Rheumatoid Arthritis Disease Activity Index (RADAI), Rapid Assessment of Disease Activity in Rheumatology (RADAR), Patient Reported Outcomes Measurement Information System (PROMIS).

Unfortunately, too many choices can paralyze a clinician into indecision, and choosing too many may increase patient burden to provide data that is not useful for their care. Therefore, it is imperative to identify the truly meaningful PROs that physicians prioritize and can act upon once collected. By doing so, they are more likely to incorporate this information into their clinical practice. In this study, we aim to understand which clinical data rheumatologists consider most important to inform treatment recommendations for their patients with RA. These results will serve as a foundation to guide selection of PROs that are most relevant, timely, and actionable to measure the relevant data.

## Methods

### Study participants

Physicians were recruited nationally by an email invitation to participate in one of 4 online nominal groups held in March and April of 2016. We sent email invitations to 325 rheumatologists who were members of the American College of Rheumatology (ACR). These members of the ACR are part of a manually curated list maintained by one of the authors (JRC). Physicians were eligible if they were rheumatologists and treated patients with RA. Invitees registered themselves to join the discussion. The investigators did not have a role in the selection of the participants for each group as invitees self-enrolled based on their availability and willingness to join the discussion. The maximum number of participants allowed to enroll per group were 12. We reached out to participants nationwide and those that participated were from the following states: Alabama, California, Florida, Montana, Pennsylvania, Puerto Rico, Tennessee, Texas, New Jersey, and New York. The University of Alabama at Birmingham Institutional Review Board approved this study. The need for consent was waived as the nominal groups were conducted online.

### Nominal group sessions

We conducted online nominal group sessions, which utilizes semi-quantitative/qualitative methodology [3–5]. Nominal groups are moderated group discussions around a single question (described below). A trained moderator led each session, assisted by a scribe. Participants called into a conference call line and logged into a website designed to support nominal group sessions. The moderator described the purpose of the study, the nominal group procedure, read and displayed on the website question for discussion. The question was: *“When seeing an established patient with RA in your office, from your personal perspective and professional practice, what elements of the patient’s history, signs, and symptoms are most helpful to you in making treatment recommendations for the management of RA?”* This question was pilot-tested with three rheumatologists and modified based on their suggestions. We excluded these three rheumatologists from participating in the nominal groups.

The moderators for each of these nominal groups were the same (MSM and ROB). Each of the four groups then generated a list of statements in response to this question. Each participant first wrote down his or her responses to the question during a 5-min silent period. We asked them to contribute a single idea expressed as a phrase or brief sentence in a round-robin format. The contributions were captured verbatim by the scribe and displayed as part of a list of contributed statements on the participants’ screens. This process was repeated until the group had consensus that all significant ideas had been captured. We reviewed and discussed all listed statements among participants to ensure that they all had a shared understanding of the statements generated as part of the discussion. Once no new statements were generated, on each of the nominal groups, participants in each group were asked to rank the statements on the list in order of importance. Each participant could vote for the three most important statements on their group’s list from their own perspective, and this rank order was retained in the software. Each nominal group session lasted approximately 90 min.

The results of each group were then aggregated into common topics by the investigators (INM, MMS, and AJ). We grouped the topics using a consensus-based process. Each group’s results were then reviewed from the perspective of the topics derived from the aggregated data.

### Analysis

In order to evaluate the contribution of statements across broader topics and analyze the results across nominal groups, individual statements were categorized into topics by investigators. The topics grouping were based on concepts stated by the participants without a prior conceptual framework. We counted the number of nominal groups that discussed each topic, the number of statements in each topic and whether any statement within a topic received any ranking vote. We created a relative importance score for each statement generated during the nominal group session to summarize the findings. Each participant had a total of six votes for the question asked in the nominal group with 3 votes for the statement selected as the most important, 2 for the second most important, 1 for the third most important, and 0 for all other statements. The number of available votes per nominal group session was dependent on the number of participants per group (e.g. 10 participants = 60 votes; 5 participants = 30 votes). We summed the votes for each statement over the participants in each group divided by the number available votes both in in each group and across groups. This resulted in each statement receiving a weighted sum of ranking votes that could be combined across groups while accounting for the differential number of individuals in each group (percentage of total votes).

## Results

Twenty-five rheumatologists participated in four groups, with 4–8 participants per group. The total of available ranking votes across all 4 nominal groups was 150 votes. Since no new ideas emerged after four groups, there was no need for further recruitment of physicians. Forty-eight percent of the participants were older than 50 years of age, 64% were women, 76% were in private practices, and 24% were from academic centers.

**Fig. 1:**
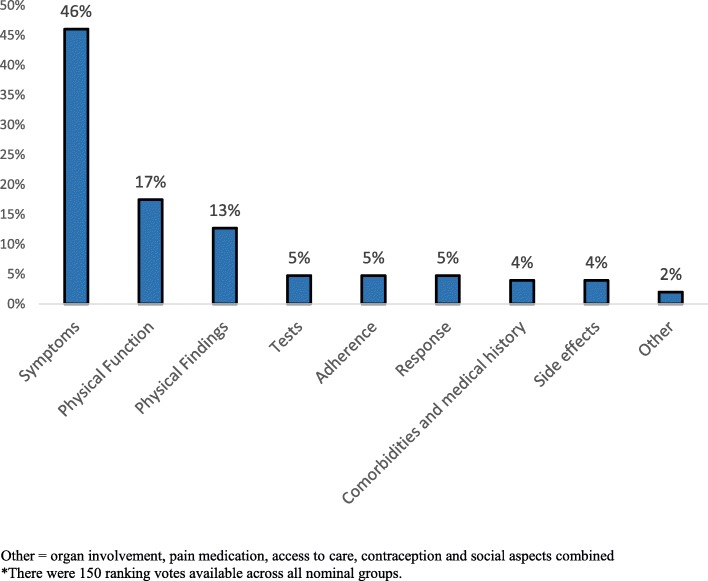
Topics that emerged during physician nominal groups and their respective percentage of votes* across all 4 groups. Other = organ involvement, pain medication, access to care, contraception and social aspects combined. *There were 150 ranking votes available across all nominal groups

“Symptoms” (defined as rheumatoid arthritis patient reported symptoms) was the topic with the highest number of votes (46%), followed by “physical function” (16%) (Fig. 1). Objective information, defined as physical findings (e.g. presence of joint swelling, tender joints) (13%) and tests (laboratory or X rays) (4%) captured 17% of the votes. Rheumatologists did not rank assessment of medication adherence highly (only 4% of votes). Among the three unranked topics (topics with statements that received 0 votes), “social aspects” (e.g. factors related to ability to work and provide for their family, family planning such as planning a pregnancy, or social support) had the highest number of statements (8 total) (See also Additional file 1: Figure S1)*.* Table 1 shows the list of the statements that physicians made during the nominal groups and within each topic and possible PROs that can capture that information.

**Table 1:** Statements and topics that rheumatologists generated across the 4 nominal groups with their respective percentage votes^a^

Statement	Percentage votes (%) (Available votes = 150)	Topics	Example of PROs Instrument
Swollen and tender joints and morning stiffness	10.0	Symptom	RADAI [6] RADAR [7]
Joints with pain, swelling and limited range of motion	9.3	Symptom	RADAI RADAR
Patient reported painful swollen joints	5.3	Symptom	RADAI RADAR
Duration of morning stiffness	4.7	Symptom	Morning stiffness duration [8]
Flare up – frequency, severity and length of flare	4.7	Symptom	OMERACT Flare core domain set [9]
How the patient feels - energy level, joint pains	2.0	Symptom	PROMIS -short form fatigue [10]
Duration of morning stiffness	2.0	Symptom	Morning stiffness duration
Increase in joint swelling	2.0	Symptom	RADAI
Features of inflammatory pain	1.3	Symptom	
Correlation between symptoms and physical examination	1.3	Symptom	
Any new symptoms or change since last visit	1.3	Symptom	
Pace of developing new symptoms	0.7	Symptom	
Fatigue compared to prior to disease onset	0.7	Symptom	PROMIS short form fatigue [10]
Sleep patterns	0.7	Symptom	PROMIS Sleep Disturbance [11]
Sleep patterns	0.0	Symptom	PROMIS Sleep Disturbance [11]
Weight loss and energy levels	0.0	Symptom	PROMIS Fatigue
Flare up resulting in visit to Emergency room	0.0	Symptom	OMERACT Flare core domain set [9]
Weight loss or weight gain	0.0	Symptom	
Description of patient’s pain	0.0	Symptom	Pain Intensity (numeric rating scale), visual analogue scale [12]
Ability to perform ADLs/HAQ	6.7	Physical Function	HAQ [13] PROMIS physical function [14]
Function/HAQ score	3.3	Physical Function	HAQ [13] MDHAQ [15]
Impact of RA on activities of interest	2.0	Physical Function	PROMIS Discretionary Social Activities [16]
Ability to participate in recreational activities	1.3	Physical Function	HAQ
Work performance and recreation	1.3	Physical Function	WPAI [17]
Change in ability to do activities	0.7	Physical Function	HAQ
Decline in functional status	0.7	Physical Function	HAQ
Ability to participate in activities	0.0	Physical Function	HAQ
Work productivity	0.0	Physical Function	WPAI [17]
Work stability	0.0	Physical Function	
Myofascial pain vs articular symptoms	0.0	Physical Function	
Presence of synovitis, number of joints, joint tenderness	4.7	Physical findings	
Tender and swollen joint count	3.3	Physical findings	
Number of swollen joints and overall mobility	2.7	Physical findings	
Number of tender joints	2.0	Physical findings	
Joint changes due to destruction or activity	0.7	Physical findings	
Articular deformities	0.0	Physical findings	
Patient assessment of disease activity	3.3	Response to treatment	
Patient feels need for medication adjustment	1.3	Response to treatment	
History of RA medication use	1.3	Response to treatment	
Adequate dosage of RA medication per patient	0.7	Response to treatment	
Improvement after change in medication	0.0	Response to treatment	
Toxicity from RA medications	2.0	Side effect	
Medication tolerance	1.3	Side effect	
Tolerance and adverse effects	1.3	Side effect	
Infections or hospitalizations	0.0	Side effect	
Inflammatory markers	2.0	Tests	
Positive rheumatoid factor and anti-CCP antibodies	2.0	Tests	
X-ray Changes	0.0	Tests	
History of bone erosions	0.0	Tests	
Compliance to current medications	2.0	Adherence	
Barriers to medication compliance	2.0	Adherence	
Issues with administration of RA medications	0.0	Adherence	
Frequency of missing RA medications	0.0	Adherence	
Compliance and regular Refilling of prescriptions	0.0	Adherence	
New or existing comorbidities	2.0	Comorbidities or medical history	
Past medical history impacting medication choices	1.3	Comorbidities or medical history	
Tuberculosis, liver disease, Congestive heart failure or malignancy	0.0	Comorbidities or medical history	
New comorbid conditions	0.0	Comorbidities or medical history	
Sleep issues or Cardiovascular risk	0.0	Comorbidities or medical history	
Smoking	0.0	Comorbidities or medical history	
Alcohol consumption	0.0	Comorbidities or medical history	
Immunization status	0.0	Comorbidities or medical history	
TB exposure	0.0	Comorbidities or medical history	
Smoking, alcohol, cholesterol levels and blood pressure	0.0	Comorbidities or medical history	
Immunization status	0.0	Comorbidities or medical history	
ILD from RA	1.3	Organ involvement	
Organ involvement from RA	0.0	Organ involvement	
Steroid dose and frequency	0.7	Pain Medication	
Opiate use	0.0	Pain Medication	
Steroid use between visits	0.0	Pain Medication	
Use of acetaminophen and NSAIDs	0.0	Pain Medication	
Use of prednisone and NSAIDs	0.0	Pain Medication	
OTC nutritional supplements	0.0	Pain Medication	
Change in financial status or health insurance	0.0	Access to Care	
Financial limitations caused by RA	0.0	Access to Care	
Finances, transportation, insurance coverage	0.0	Access to Care	
Impact of contraception on RA medication	0.0	Contraception	
Current use of contraception	0.0	Contraception	
Impact of RA on patient’s relationship	0.0	Social aspects	
Current or planning of pregnancy	0.0	Social aspects	
Patient’s ability to manage disease	0.0	Social aspects	
History of depression	0.0	Social aspects	
Hobbies/sports	0.0	Social aspects	
Family responsibilities	0.0	Social aspects	
Planning parenthood	0.0	Social aspects	
Social history and change in job	0.0	Social aspects	

^a^Note: Each of the statements listed here were generated in the nominal groups. Few participants may have mentioned the same statement in different groups and they were added here with the respective percentage vote that receive in each particular group

*HAQ* Health assessment questionnaire [13], *ADLs* Activities of daily living, *Anti-CCP* Anti-Cyclic Citrullinated Peptide, *TB* Tuberculosis, *ILD* Interstitial lung disease, *NSAIDs* Non-steroidal anti-inflammatory drugs, *OTC* Over the counter

*OMERACT* Outcome Measures in Rheumatology

*RADAI* Rheumatoid Arthritis Disease Activity Index [6]

*RADAR* Rapid Assessment of Disease Activity in Rheumatology [7]

*PROMIS* Patient Reported Outcomes Measurement Information System [10, 11, 14, 16]

*WPAI* Work productivity and activity impairment Questionnaire [17]

**Fig. 2:**
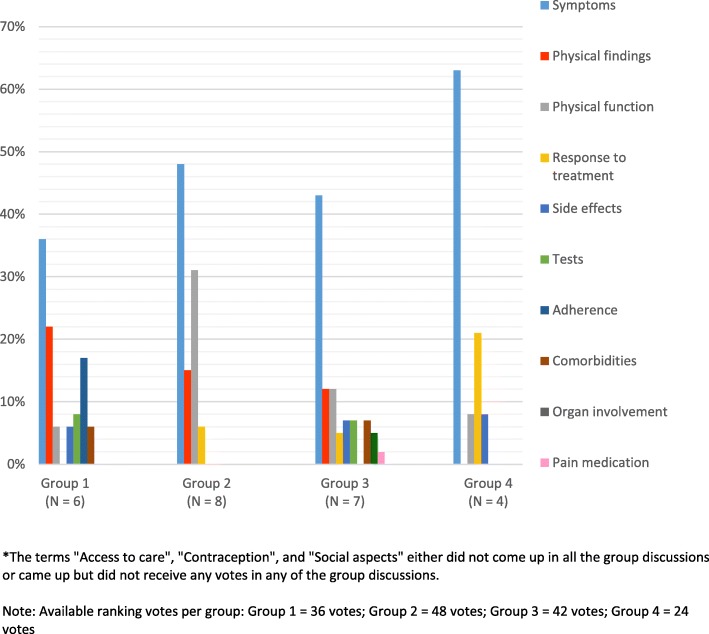
Topics that emerged from four nominal groups of rheumatologists and their respective percentage of total votes, by group *The terms “Access to care”, “Contraception”, and “Social aspects” either did not come up in all the group discussions or came up but did not receive any votes in any of the group discussions. Note: Available ranking votes per group: Group 1 = 36 votes; Group 2 = 48 votes; Group 3 = 42 votes; Group 4 = 24 votes

Of the 13 topics that emerged from the aggregated data, 10 received ranking votes with 9 of these topics been mentioned in either 3 or 4 of the groups (Table 1). Additional file 2: Table S1 shows the counts of 1st ranked, 2nd ranked, and 3rd ranked statements. The topics with the highest number of ranking votes within each group were “symptoms”. “Physical findings” received the second highest number of votes in group 1 and 3, and third highest in group 2 (Figs. 1 and 2). The percentage of votes in groups 2 and 4 were distributed across only 4 topics, with “symptoms” receiving a high proportion of the votes in each of these groups. Group 1’s priorities were distributed across 7 topics, and group 3’s across 9 topics, with “symptoms,” “physical findings”, and “physical function” receiving the most votes.

## Discussion

Patient-reported information most valued by the rheumatologists in our study were rheumatoid arthritis-related symptoms and physical function. Objective information such as physical examination findings was important as well. They enquired on other aspects of life such as family planning, social support, relationship with their spouse, and depression. Even though these “social aspects” did not receive ranking votes, it was the topic with the greatest number of statements among the unranked topics, suggesting that quantitative assessment of this topic may also be of value for practicing rheumatologists.

The most valued among symptoms, was patient-reported joint swelling, tenderness, and morning stiffness. These symptoms can be captured by using patient-reported instrument such as RADAI, which constitutes global disease activity in the last 6 months, current disease activity in terms of swollen and tender joins, pain, duration of morning stiffness and tender joints on a list [6]. RADAR questionnaire contains global disease activity the past 6 months, current disease activity in terms of joint tenderness and swelling, pain, duration of morning stiffness, functional class and a tender joint list and can measure above mentioned statements as well [7]. Morning stiffness duration is also a component of the RADAI and RADAR questionnaires and there is an ongoing effort to improve measurement of stiffness by OMERACT (Outcome Measures in Rheumatology) [18]. Other elements of symptoms such as fatigue, sleep, and physical function can be quantified using PROMIS and HAQ, respectively. Additionally, inquiring about fatigue, depression, and sleep patterns is evidence of awareness among study physicians about these coexisting conditions. There is growing literature that RA is associated with comorbidities such as depression, anxiety, fatigue, and sleep disorders [19–21]. These problems may not be directly related to RA but are often associated with poor clinical outcomes and increased disease activity scores [22]. Recognition of these comorbidities with PROs can help early management and improve patient’s quality of life.

Shared decision making between the patient and physician on the treatment of rheumatoid arthritis is advocated by the clinical practice guidelines [23]. To accomplish the goals of shared-decision making, priorities from both, patients and physicians should be met. Previous studies show that there are differences between patients and physicians in terms of perspectives and priorities in management of RA. For example, discordance is noted between patient and physician global assessment of RA disease activity in approximately one third of cases [24]. Physicians are more likely to rate RA disease activity lower than patients, possibly because they rely more on objective measures. However, patients have a more holistic view of their health that does not excessively partition RA disease activity distinctly from other related impact on their health and consider physical function, fatigue and health related quality of life as important factors [25, 26]. PROs describe a patient’s health status from their own perspective thereby providing valuable complementary information to an assessment focused only on RA and allow physicians to assess health in domains that patients consider important so as to provide more holistic care.

Despite the utilization of PROs in the research studies and trials, their incorporation into clinical practice is hampered by multiple challenges. A survey of 439 rheumatologists in the United States showed that they were not opposed to use quantitative measurement such as PROs while assessing patients with RA, but many clinicians felt that they lacked the time and electronic tools to do so efficiently [27]. Patient-related factors such as health literacy, psychological stress, language proficiency may impact both the collection of and the utilization of PROs in real world settings [28]. There is also a lack of understanding by many clinicians on how to effectively use the data over time, and to interpret changes in PROs. For example, analyses comparing RA treatment changes in response to moderate or high disease activity as measured by the RAPID3 vs. the clinical disease activity index (CDAI) suggests that the a high CDAI score prompt RA treatment changes more so than RAPID3 [29]. The lack of psychometric assessment and validation of PROs in specific population (e.g. patients with high comorbidity burdens) is another limitation. Finally, while some PROs are commonly collected, such as the Patient global assessment (PGA), patients may feel that the PROs commonly measured in RA lack some relevance while more important health domains may be neglected (e.g ability to participate in meaningful social relationships; impairment in work-related activities etc.) [30]. Our previous work showed that patients lost interest in completing PROs if the rheumatologist did not use the provided information [31]. Thus, in the present study, we attempted to identify the information most valued by the rheumatologists to make treatment recommendations for RA so we can further determine which PROs could be utilized to measure it. We were able to generate a list of statements that we can now put together into a repository of PROs for electronic data collection. In this way, rheumatologists could utilize this data, as it is something they value and could also motivate patients to complete PROs because their physician is likely to act on it.

We will use the results of our hypothesis-generating study to devise a quantitative assessment of a representative sample of rheumatologists to confirm their priorities expressed by the sample in our study. Additionally, assessment of their preferences on how frequently and in what form (e.g. tablet, computer, paper, embedded in the electronic health record) they would prefer to collect this data will be needed. The strengths of this study were the use of the nominal group technique to collect semi-quantitative data. Studies suggest that this technique is suited for the development of multiple perspectives on an issue, elicitation of responses without strong opinions or personalities (a limitation of qualitative research such as focus groups), and prioritizing root causes of a problem [3, 32]. This method has demonstrated validity, and considers all participants’ views equally [33]. A high degree of agreement on responses from the different groups in our study met our research needs and re ranking was not considered [34–36]. No new ideas emerged towards the end of discussion, which further contributed to the strength of the data. We recruited rheumatologists nationally, which reflects clinical practices throughout the nation and not only regionally.

The study limitations were that the nominal group technique does not allow development of full discussion and therefore the attitudes and beliefs of the participants were not fully elicited. It is possible that the distribution of physicians (and their preferences) could have affected the distribution of the topics. The characteristics of physicians were not identified until they had completed the session. We did not determine if either an academic physician, a private practitioner, early career, or senior career physician made particular statements. Therefore, we could not ascertain what factors may have influenced the voted topics as it was beyond the scope of nominal group session. The hypothesis generating stage of the research also precludes the generalizability to a larger population of rheumatologists, thus it is possible that a larger sample survey could result in different priorities. We note that this was not the goal of the research, lessening this concern, and that our study forms the foundation for future work in this important area.

## Conclusions

Rheumatologists value certain aspects related to RA to inform their treatment recommendations that can be measured through PROs. The results of this study will be used to develop a survey for rheumatologists to assess the generalizability of the findings. This research will serve as a foundation for the development of a user-friendly PROs data collection platform for the physicians to aid treatment decisions in RA. Such tools can facilitate the data collection for RA registries, implementation of the treat to target guidelines, and optimization of clinical care for patients with RA.

## Additional files

Additional file 1: **Figure S1.** Topics that Emerged During Physician Nominal Groups and the Respective Number of Participants that Voted for the Statements within Each Topic as the 1st, 2nd, and 3rd most important. (DOCX 42 kb)

Additional file 2: **Table S1.** Statements with the respective distribution of votes as the 1st, 2nd, and 3rd most important. (DOCX 19 kb)

## Data Availability

All data generated or analyzed during this study are included in this published article and its supplementary information files.

## Abbreviations

ADLs: Activities of daily living
Anti-CCP: Anti-Cyclic Citrullinated Peptide
CDAI: Clinical Disease Activity Index
HAQ: Health assessment questionnaire
ILD: Interstitial lung disease
MDHAQ: Multi-Dimensional Health Assessment Questionnaire
NSAIDs: Non-steroidal anti-inflammatory drugs
OTC: Over the counter
PROMIS: Patient-Reported Outcomes Measurement Information System
PROs: Patient-reported outcomes
RA: Rheumatoid arthritis
RADAI: Rheumatoid Arthritis Disease Activity Index
RADAR: Rapid Assessment of Disease Activity in Rheumatology
RAPID3: Routine assessment of patient index data 3
TB: Tuberculosis
WPAI: Work productivity and Activity impairment questionnaire
