# Supplementary material for: Which patient reported outcome domains are important to the rheumatologists while assessing patients with rheumatoid arthritis?
Source: BMC Rheumatol. 2019 Sep 5;3:36. doi: 10.1186/s41927-019-0087-2 (PMC6727422; doi:10.1186/s41927-019-0087-2)

***Additional file 1: Figure S1:*** Topics that Emerged During Physician Nominal Groups and the Respective Number of Participants that Voted for the Statements within Each Topic as the 1^st^, 2^nd^, and 3^rd^ most important.


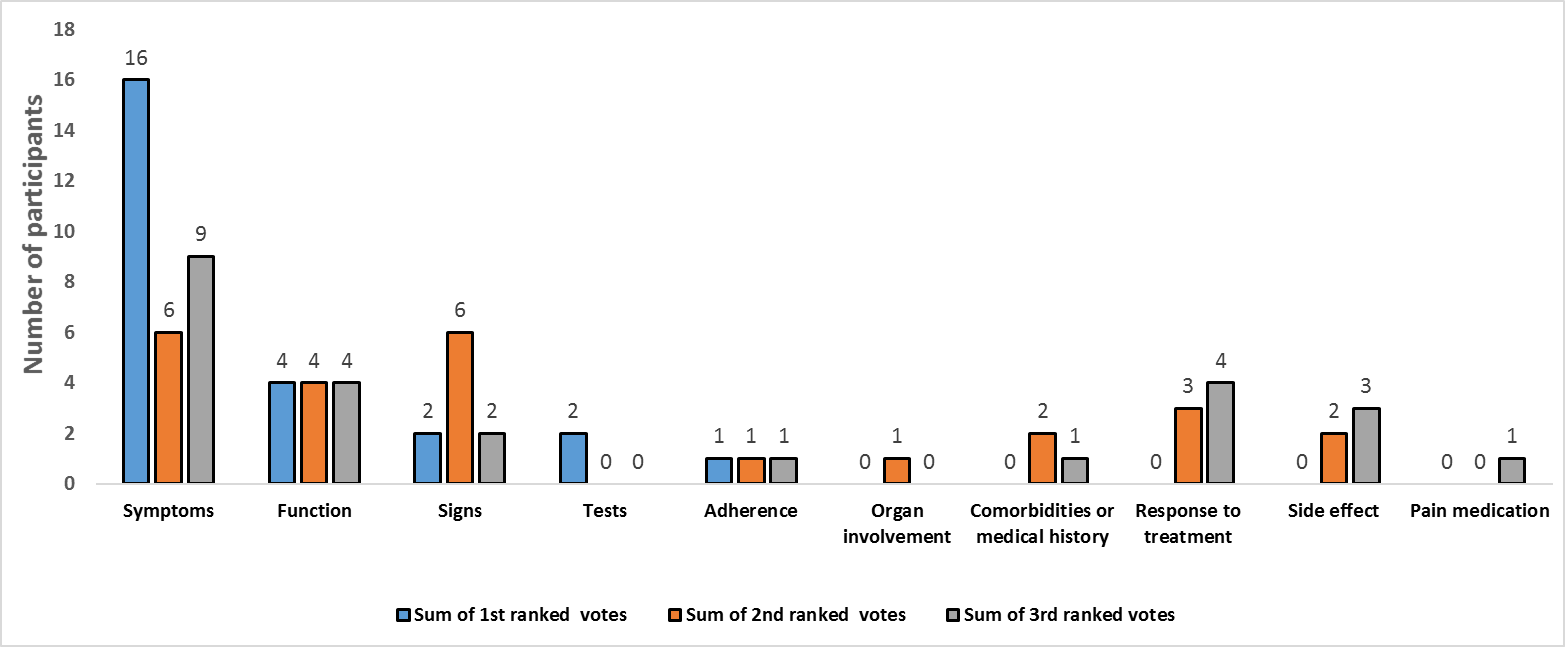

Supplement: Supplementary file 1 — Figure S1. Topics that Emerged During Physician Nominal Groups and the Respective Number of Participants that Voted for the Statements within Each Topic as the 1st, 2nd, and 3rd most important. (DOCX 42 kb) [file 41927_2019_87_MOESM1_ESM.docx]
