# Supplementary material for: Which patient reported outcome domains are important to the rheumatologists while assessing patients with rheumatoid arthritis?
Source: BMC Rheumatol. 2019 Sep 5;3:36. doi: 10.1186/s41927-019-0087-2 (PMC6727422; doi:10.1186/s41927-019-0087-2)
Supplement: Supplementary file 2 — Table S1. Statements with the respective distribution of votes as the 1st, 2nd, and 3rd most important. (DOCX 19 kb) [file 41927_2019_87_MOESM2_ESM.docx]

**Additional file 2: Table S1:** Statements with the respective distribution of votes as the 1^st^, 2^nd^, and 3^rd^ most important.

| **Group** | **Topic** | **Statement** | **Total score** | **#1 vote** | **#2 vote** | **#3 vote** |
| --- | --- | --- | --- | --- | --- | --- |
| 2 | Symptom | Swollen and tender joints and morning stiffness | 15 | 4 | 0 | 3 |
| 3 | Symptom | Joints with pain, swelling and limited range of motion | 14 | 4 | 1 | 0 |
| 4 | Symptom | Patient reported painful swollen joints | 8 | 2 | 1 | 0 |
| 1 | Symptom | Duration of morning stiffness | 7 | 2 | 0 | 1 |
| 4 | Symptom | Flare up – frequency, severity and length of flare | 7 | 2 | 0 | 1 |
| 2 | Symptom | How the patient feels - energy level, joint pains | 3 | 1 | 0 | 0 |
| 3 | Symptom | Duration of morning stiffness | 3 | 0 | 1 | 1 |
| 1 | Symptom | Increase in joint swelling | 3 | 1 | 0 | 0 |
| 1 | Symptom | Features of inflammatory pain | 2 | 0 | 1 | 0 |
| 2 | Symptom | Correlation between symptoms and physical examination | 2 | 0 | 1 | 0 |
| 2 | Symptom | Any new symptoms or change since last visit | 2 | 0 | 1 | 0 |
| 1 | Symptom | Pace of developing new symptoms | 1 | 0 | 0 | 1 |
| 2 | Symptom | Fatigue compared to prior to disease onset | 1 | 0 | 0 | 1 |
| 3 | Symptom | Sleep Patterns | 1 | 0 | 0 | 1 |
| 2 | Physical Function | Ability to perform ADLs/HAQ | 10 | 2 | 2 | 0 |
| 3 | Physical Function | Function/HAQ score | 5 | 1 | 1 | 0 |
| 2 | Physical Function | Impact of RA on activities of interest | 3 | 1 | 0 | 0 |
| 2 | Physical Function | Ability to participate in recreational activities | 2 | 0 | 0 | 2 |
| 4 | Physical Function | Work performance and recreation | 2 | 0 | 1 | 0 |
| 1 | Physical Function | Change in ability to do activities | 1 | 0 | 0 | 1 |
| 1 | Physical Function | Decline in functional status | 1 | 0 | 0 | 1 |
| 1 | Physical Findings | Presence of synovitis in number of joints, joint tenderness | 7 | 1 | 2 | 0 |
| 3 | Physical Findings | Tender and swollen joint count | 5 | 1 | 1 | 0 |
| 2 | Physical Findings | Number of swollen joints and overall mobility) | 4 | 0 | 2 | 0 |
| 2 | Physical Findings | Number of tender joints | 3 | 0 | 1 | 1 |
| 1 | Physical Findings | Joint changes due to destruction or activity | 1 | 0 | 0 | 1 |
| 4 | Response to treatment | Patient assessment of disease activity e | 5 | 0 | 2 | 1 |
| 2 | Response to treatment | Patient feels need for medication adjustment | 2 | 0 | 1 | 0 |
| 3 | Response to treatment | History of RA medication use | 2 | 0 | 0 | 2 |
| 2 | Response to treatment | Adequate dosage of RA medication per patient | 2 | 0 | 0 | 1 |
| 3 | Side effect | Toxicity from RA medications | 3 | 0 | 1 | 1 |
| 1 | Side effect | Medication tolerance | 2 | 0 | 1 | 0 |
| 4 | Side effect | Tolerance and adverse effects | 2 | 0 | 0 | 2 |
| 1 | Tests | Inflammatory markers | 3 | 1 | 0 | 0 |
| 3 | Tests | Positive rheumatoid factor and anti-CCP antibodies | 3 | 1 | 0 | 0 |
| 1 | Adherence | Compliance to current medications | 3 | 0 | 1 | 1 |
| 1 | Adherence | Barriers to medication compliance | 3 | 1 | 0 | 0 |
| 3 | Comorbidities or medical history | New or existing comorbidities | 3 | 0 | 1 | 1 |
| 1 | Comorbidities or medical history | Past medical history impacting medication choices | 2 | 0 | 1 | 0 |
| 3 | Organ involvement | Interstitial lung disease from RA | 2 | 0 | 1 | 0 |
| 3 | Pain medication | Steroid dose and frequency | 1 | 0 | 0 | 1 |

Note: The statements within each topic that did not receive any votes are excluded from this table. Access to care and contraception did not receive any votes
